# Orthorexic Tendency and Eating Disorders Symptoms in Polish Students: Examining Differences in Eating Behaviors

**DOI:** 10.3390/nu12010218

**Published:** 2020-01-15

**Authors:** Marta Plichta, Marzena Jezewska-Zychowicz

**Affiliations:** Department of Food Market and Consumer Research, Institute of Human Nutrition Sciences, Warsaw University of Life Sciences (SGGW-WULS), Nowoursynowska 159C, 02-776 Warsaw, Poland; marzena_jezewska_zychowicz@sggw.pl

**Keywords:** orthorexic tendency, eating disorders symptoms, eating habits, dietary patterns, students

## Abstract

Orthorexia nervosa (ON) may precede, ensue, or coexist with eating disorders (ED) and also affect eating behaviors. The aim of this study was to explore the dietary patterns (DPs) and other eating habits of people showing ON tendency, ED symptoms, and both ON tendency and ED symptoms, as well as those not showing either. The data for the study were collected from a sample of 1120 Polish college students through a cross-sectional survey in 2017. The questionnaire used in the survey included the ORTO-15, the Eating Disorder Screen for Primary Care (ESP), and the Food Frequency Questionnaire (FFQ-6), and the students were also asked questions about their eating habits and special diets. A factor analysis was conducted to identify the following five DPs: “high-sugar products and refined products,” “meat and meat products,” “alcohol,” “high-fiber products and nuts,” and “dairy products and whole-meal bread.” Univariate logistic regression analyses were carried out to verify the associations between the variables. Students in the “ON and without ED” group were found to exhibit more healthy eating habits than the students in the “ED and without ON” group. The use of a special diet in the past and currently increased the risk of displaying ON tendency and ED symptoms. Students in the “ON and without ED” and “ON and ED” groups were characterized by less frequent consumption of high-sugar and refined products. Students who rarely consumed meat and related products were found to be less likely to display “ON and ED.” In conclusion, different eating habits shown by people with ON tendency and ED symptoms confirmed the difference between ON and ED. However, the simultaneous displaying of ON and ED leads to the overlapping of specificity of eating habits, which can make the diagnosis based only on the eating habits difficult. Thus, there is still a need for further research involving the use of more sensitive tools that can better identify ON tendency and ED symptoms, as well as DPs.

## 1. Introduction

Orthorexia nervosa (ON) is defined as the obsession with eating only healthy food, which includes specific attitudes toward the planning, choice, purchase, preparation, and consumption of food [1]. Individuals with ON focus more on a perfect, pure, and healthy diet, which may have both preventive and healing effects on certain illnesses [2]. Nevertheless, such an obsessive behavior can lead to harmful consequences similar to those of eating disorders (ED) [3].

Considerable controversies exist regarding the classification of ON as some researchers suggest that ON is similar to the currently recognized ED, obsessive-compulsive disorders (OCD), somatoform disorders, and autism spectrum disorders [4,5]. However, so far, no diagnostic criteria have been proposed for orthorexia [3,6], and ON is also not included in the Diagnostic and Statistical Manual of Mental Disorders (DSM-5) [7].

The association between ON and ED is not unequivocal. A study by Segura-Garcia et al. [8] showed that 28% of patients with anorexia nervosa (AN) and bulimia nervosa (BN) were found to exhibit ON tendency. Additionally, the proportion of patients displaying ON tendency increased to 53% at the end of the ED treatment. These findings suggest that ON may precede, ensue, or coexist with ED [8]. However, ON does not include the primary symptoms of AN or BN, such as the fear of being obese, an excessive preoccupation with one’s body, and a distorted perception of body image [9]. Moreover, a previous study reported that individuals with ON tendency expressed greater satisfaction with their upper body [10].

ON shares some similarities with OCD, such as intrusive thoughts of food and health and compulsive behaviors while preparing and eating food [4]. However, the obsessions observed in individuals with OCD are perceived as ego-dystonic, while those observed in people with ON are perceived as ego-syntonic [9]. Additionally, questions have been raised as to whether ON can be classified in the same category as avoidant/restrictive food intake disorder (ARFID). However, ARFID and ON have an important difference; in the former, abnormal eating behaviors result from food anxiety or fear, whereas in the latter, such behaviors occur due to excessive concerns for health [11].

In addition to the lack of a universally shared definition and diagnostic criteria, there are no specific tools for evaluating ON tendency. The psychometric instruments that have been used in the studies on ON include the Bratman Orthorexia Test (BOT) [1] and the ORTO-15 [12], but these have revealed some methodological flaws [13,14]. However, the original version of ORTO-15 has been validated, adapted to other languages, and used in research on ON in some countries [15,16,17,18].

The findings of some studies suggest that most of the strictly followed diets may lead to ON [1]. The prevalence of ON is higher among individuals who follow a vegetarian, vegan, fruitarian, macrobiotic, or raw food diet [19,20] and among those who are involved in the animal welfare organizations and the supporters of organic and nongenetically modified foods [21]. ON is also related to healthy lifestyle choices, such as higher intake of fruits and vegetables, lower intake of white cereals, shopping only in healthy food stores, and reduced consumption of alcohol [18].

Previous studies that analyzed the occurrence and symptoms of ON have focused on the eating behaviors to some extent. The researchers first assessed ON tendency in groups following special diets, especially a vegetarian or vegan diet [22,23,24,25], and evaluated the frequency of food intake [26]. To date, other eating habits or dietary patterns (DPs) of people showing ON tendency have only been rarely studied [10,27]. Moreover, knowledge about the differences in the eating behaviors of people with ON and ED is insufficient to confirm both the similarities and differences between these two conditions. Therefore, the aim of this study was to explore the DPs identified based on the frequency of food consumption and other eating habits in people showing ON tendency, ED symptoms, and both ON tendency and ED symptoms, as well as those not showing both. We examined the eating habits of the participants from their responses to questions on the number of meals consumed, the regularity of meal consumption, skipping of meals, and following a special diet.

## 2. Materials and Methods

### 2.1. Ethical Approval

The study was approved by the Ethics Committee of the Faculty of Human Nutrition and Consumer Science, Warsaw University of Life Sciences (Resolution Number 45/2017). Informed consent was obtained from all the participants included in the study.

### 2.2. Study Design and Sample Collection

The study sample was recruited from seven universities, which were located in northern, central, and southern Poland. A survey was conducted in the universities by a trained interviewer in 2017. The process was carried out during lectures, and the instructors left the lecture rooms after consenting to the study. The students were informed that participation in the study was voluntary. A total of 1300 students of health-related and other majors agreed to participate in the study. The study sample included female and male students aged 18–35 years old. The participants were informed about the purpose, mileage, and duration of the study, as well as that they can withdraw from the study at any time without any consequences. Due to the lack of data in the questionnaire, 162 participants were excluded from the sample. Eighteen students were older than 35 years old and were therefore also excluded. The final sample consisted of 1120 students.

### 2.3. Orthorexia Nervosa Tendency

To assess the ON tendency, we used the Polish version of the ORTO-15 questionnaire [16]. The reliability values of the Polish version of this questionnaire suggested a good internal consistency (Cronbach’s alpha 0.7–0.9) [16]. The ORTO-15 is a 15-item questionnaire based on a Likert scale (always, often, sometimes, never) that aims to examine the rational, cognitive, and emotional symptoms of ON [12]. The items in the questionnaire that indicate ON tendency are scored as 1, while those describing adequate eating behaviors are scored as 4. Thus, the total score of the scale can range from 15 to 60.

### 2.4. Eating Disorders Symptoms

The Polish version of the Eating Disorder Screen for Primary Care (ESP) questionnaire [28] was used to identify the ED symptoms. The ESP is a short 5-item screening instrument. Answers “no” to question 1 or “yes” to the other four questions indicate an abnormal answer which in turn indicates that individuals need further assessment [28].

### 2.5. Eating Habits

Eating habits of the participants were assessed based on the following questions: “How many meals do you eat during a day?” (answers: “1–2 meals,” “3 meals” or “4 and more meals”), “Do you eat meals regularly?” (answers: “yes,” “no”), “How long are the breaks between your meals?” (answers: “less than 3 h,” “3–4 h” or “more than 4 h”), “Do you eat breakfast/dinner/supper?” (answers: “yes,” “no”), “Do you follow currently/in the past a special diet?” (answers: “yes,” “no”).

### 2.6. Frequency of Food Consumption

Dietary data were collected from the participants using a validated Food Frequency Questionnaire (FFQ-6) [29]. The FFQ-6 tool consists of a list of 62 food items and evaluates the frequency of food consumption over the last 12 months. The questionnaire includes products and dishes representing 8 major food groups such as sweets and snacks, dairy products and eggs, cereal products, fats, fruits, vegetables and grains, meat and fish products, and beverages. The following categories were used to indicate the frequency of food consumption: 1—Never or almost never, 2—Once a month or less frequently, 3—Several times per month, 4—Several times per week, 5—Every day, and 6—Several times per day.

### 2.7. Sociodemographic Characteristics

Information on sociodemographic characteristics was also collected from the participants during the survey, which included gender, age (in years), place of residence (village, town with 100,000 inhabitants or less, city with more than 100,000 inhabitants), level of education (bachelor and engineering or master degree programs), college major (health or nonhealth related), and self-reported weight (in kg) and height (in cm). Body mass index (BMI) was calculated (in kg/m^2^), and the values were categorized into four groups according to the guidelines of the World Health Organization [30]: underweight (BMI < 18.5 kg/m^2^), normal weight (18.5 ≥ BMI ≤ 24.9 kg/m^2^), overweight (25.0 ≥ BMI ≤ 29.9 kg/m^2^), and obesity (BMI ≥ 30.0 kg/m^2^).

### 2.8. Statistical Analysis

Descriptive statistics, including frequency distributions and cross-tabulations, were performed for the data. DPs were identified from the factor analysis based on the frequency of food consumption. The factors were rotated by an orthogonal (varimax) transformation. The number of factors was determined based on the following criteria: components with an eigenvalue of 1, a scree plot test, and the interpretability of the factors. The eigenvalues signify the amount of variance explained by each of the factors. Dietary variables with factor loadings of at least 0.50 were taken into account. The factorability of the data was confirmed with the Kaiser–Meyer–Olkin (KMO) measure of sampling adequacy and Bartlett’s test of sphericity. The KMO value was found to be 0.803, and Bartlett’s test was significant at *p* < 0.0001 [31]. Five DPs (factors) were derived: “high-sugar products and refined products,” “meat and meat products,” “alcohol,” “high-fiber products and nuts,” and “dairy products and whole-meal bread.” The total variance explained was 54.8%. The explained variance for the factors was 13.6%, 11.5%, 11.4%, 9.4%, and 8.9%, respectively. The factor-loading matrix for the DPs identified by the factor analysis is presented in detail in Table 1.

Based on the tertile distribution, participants were divided into three groups within each DP (bottom, middle, or upper tertile). The upper tertile represented the greatest adherence to the DP, while the bottom represented the lowest adherence.

According to the authors of the original version of ORTO-15 [12], a score below 40 indicates ON tendency, whereas higher scores indicate adequate eating behaviors. There has been some discussion as to whether a cut-off of 40 is too high and a lower cut-off of 35 should be used [32,33,34]. Therefore, in our study, we used the cut-off of 35. Cronbach’s alpha for ORTO-15 was 0.7.

One or no abnormal response to the questions in the ESP questionnaire [28] ruled out the ED symptoms, while two or more abnormal answers showed the presence of ED symptoms and indicated the need for further diagnosis of ED in the patients.

Respondents were divided into 4 groups based on their responses to the ORTO-15 and the ESP questionnaire: 1/people displaying ON tendency but not ED symptoms (“ON and without ED”), 2/people displaying ED symptoms but not ON tendency (“ED and without ON”), 3/people displaying both ON tendency and ED symptoms (“ON and ED”), and 4/people displaying neither ON tendency nor ED symptoms (“neither ON nor ED”). 

The normality of the variable distribution was examined with a Shapiro–Wilk test. The differences between groups were verified by a chi-squared test (gender versus identified groups of participants (“ON and without ED,” “ED and without ON,” “ON and ED,” “neither ON nor ED”) and eating habits).

Univariate logistic regression analyses were carried out to verify the associations between the groups of participants identified according to the responses to the ORTO-15 and ESP questionnaires (“ON and without ED,” “ED and without ON,” “ON and ED,” “neither ON nor ED”) and based on eating habits and DPs and as following a special diet. The odds ratios (ORs) and 95% confidence intervals (95% CIs) were calculated. ORs represented the chances of adherence of the participants to the individual DPs taking into account the occurrence of ON tendency and ED symptoms. The reference groups were students eating four or more meals a day, eating meals regularly, eating meals every 3–4 h, skipping breakfast, dinner, and supper, and following a special diet currently or in the past, as well as those who represented the upper tertile of each DP (OR = 1.00). The significance of ORs was assessed by Wald’s statistics. The level of statistical significance was set at *p* < 0.05. The statistical analysis was carried out using Statistica software version 13.3 (StatSoft Inc., Tulsa, OK, USA; StatSoft, Krakow, Poland).

## 3. Results

### 3.1. Sample Characteristics

The sociodemographic characteristics of the study participants are summarized in Table 2. The study sample included 70.4% females and 29.6% males. More than 70% of the participants were under 23 years of age. Around 48.8% of the participants were studying health-related majors (food technology and human nutrition, dietetics, physiotherapy, physical education, and wellness), while 51.2% were studying other majors. Most of the participants were enrolled in bachelor and engineering degree programs (88.7%). More than two-fifths of the participants lived in large cities (43.1%). About three-quarters of the participants (72.9%) had a normal weight.

### 3.2. Orthorexia Nervosa Tendency, Eating Disorders Symptoms, and Eating Habits across Gender

ON tendency was found in 28.3% of the study sample, while ED symptoms were found in 50.7%. After grouping the respondents, including under the ON and ED categories, it was found that more than one-third of them (37.7%) displayed only ED symptoms (“ED and without ON”), while 15.3% exhibited only ON tendency (“ON and without ED”). Both ON tendency and ED symptoms (“ON and ED”) were identified in 13% of the study sample, while their incidence was not found in 34%. In the study sample, more females displayed only ED symptoms (“ED and without ON”) compared to males (*p* < 0.0001), while more males displayed neither ON tendency nor ED symptoms compared to females (*p* < 0.0001). No significant gender differences were observed for the other categories (“ON and without ED” and “ON and ED”) (Table 3).

Nearly two-thirds of the participants reported that they consumed four or more meals a day (61.1%), while more than half did not eat meals regularly (58.7%); however, no gender differences were observed for these eating habits. Almost 70% of the students declared eating meals every 3–4 h. More females (71.6%) declared eating meals every 3–4 h compared to males (62.3%), whereas more males (32.0%) declared having a break of more than 4 h between meals compared to females (21.9%) (*p* = 0.001). Most of the students reported that they did not skip breakfast (85.6%), dinner (92.3%), and supper (87.9%). Moreover, more than two-fifths of the participants reported having followed a special diet in the past (41.9%), while only 12.6% of the students reported as following a special diet currently. No gender differences were observed for skipping meals and following a special diet currently or in the past (Table 3).

### 3.3. Associations between Orthorexia Nervosa Tendency, Eating Disorders Symptoms, Eating Habits, and Dietary Patterns

Table 4 presents the results of the univariate logistic regression analyses showing the associations between the groups of participants identified with ON tendency and ED symptoms, their eating habits, and DPs. The results demonstrated that the students who consumed 1–2 or three meals a day were less likely to display “ON and without ED” (ORs: 0.24 and 0.48, respectively) compared to those who consumed four or more meals a day. Moreover, the students who reported eating meals irregularly were less likely to display “ON and without ED” than those eating meals regularly (OR: 0.30). In the case of students having longer than 4 h of break between meals, the risk of “ON and without ED” was 58% lesser (OR: 0.42) compared to those eating meals every 3–4 h. Students who reported not skipping dinner and supper were more likely to display “ON and without ED” (ORs: 6.33 and 2.70, respectively) than those skipping these meals. Moreover, the risk of “ON and without ED” in the students who did not currently follow a special diet was lower (OR: 0.36) compared to those currently following such a diet. Students who rarely consumed “High-sugar products and refined products” (bottom tertile of the DP) were more likely to display “ON and without ED” (OR: 1.66) compared to those who frequently consumed such products (upper tertile of the DP).

Students who consumed 1–2 or three meals a day were more likely to display only ED symptoms (“ED and without ON”) compared to those who consumed four or more meals a day (ORs: 2.24 and 1.40, respectively). Similarly, the students who declared eating meals irregularly and having a break longer than 4 h between meals were more likely to show only ED symptoms (“ED and without ON”) compared to those eating meals regularly and having 3–4 h of break between meals (ORs: 2.35 and 1.49, respectively). By contrast, the students who reported not skipping dinner and supper were less likely to display only ED symptoms (“ED and without ON”) compared to those skipping these meals (ORs: 0.56 and 0.59, respectively). In addition, the students who reported as not having followed a special diet in the past or following such a diet currently were less likely to display only ED symptoms (“ED and without ON”) compared to those who followed such a diet (ORs: 0.74 and 0.40, respectively). No significant associations were observed between displaying only ED symptoms (“ED and without ON”) and the identified DPs (Table 4).

The risk of displaying both ON tendency and ED symptoms (“ON and ED”) was lower in students who did not follow a special diet in the past or currently (ORs: 0.34 and 0.26, respectively) than those who followed such a diet. No significant associations were observed between “ON and ED” and other eating habits. Students in the bottom tertile of the DP “high-sugar products and refined products” were more likely to display “ON and ED” (OR: 1.67) compared to those in the upper tertile of this DP. Moreover, students who rarely consumed meat and meat products (bottom tertile of the DP “meat and meat products”) were less likely to display “ON and ED” (OR: 0.58) than those in the upper tertile of this DP.

It was found that the students who did not follow a special diet in the past or currently were more likely to be in the category “neither ON nor ED” (ORs: 2.69 and 2.65, respectively) compared to those who followed such a diet. Students who rarely consumed high-sugar and refined products (the bottom tertile of the DP) were less likely to display ON tendency and ED symptoms (“neither ON nor ED”) compared to those in the upper tertile of the DP “high-sugar products and refined products” (OR: 0.66). No significant associations were found for the DP “neither ON nor ED” with other DPs or eating habits, except following a special diet.

## 4. Discussion

In this study, we assessed the occurrence of ON tendency and ED symptoms in a large group of Polish students studying various majors. By applying the ORTO-15 questionnaire and a cut-off age of 35, ON tendency was identified in 28.3% of the study sample, which was consistent with the results of other studies using this cut-off point [33,35]. According to some researchers, lowering the cut-off from the recommended score of 40 to 35 allows for better identification of the ON tendency [32,33,34].

In our study, it was found that almost half of the people with the ON tendency also displayed ED symptoms. The relationship between the ON tendency and ED symptoms is complex and not clear enough and hence needs to be studied in detail [36]. In some studies, ON was indicated as a predictor of ED. The risk of developing ON tendency was five-fold higher in ED patients compared to healthy people [37]. Although ON tendency may precede ED, it may also occur during the remission phase of ED [36]. While some researchers consider ED as a risk factor for ON tendency [8], ED is also pointed out as a negative predictor of ON [38]. Although our cross-sectional research did not allow understanding the causal relationship between ON tendency and ED symptoms, their coexistence in 13% of the study sample, which included half of the students who showed ON tendency, confirmed the relationship between ON and ED.

In the present study, we noted that a high proportion of people were showing only ED symptoms (37.7%) in our study sample. In other studies conducted among students in nine countries, the incidence of ED symptoms ranged from 2.2% to 29.1% [39]. This finding of a larger percentage of Polish students with ED symptoms compared to the other studies included in the meta-analysis may have resulted from the use of a different tool for assessing ED symptoms [39]. The ESP questionnaire used in our study [28] is a self-descriptive tool that measures only the symptoms of ED but does not allow clinical diagnosis. Although such tools have limitations, they are useful for monitoring subclinical or threshold ED symptoms [39]. The rates of occurrence of ED symptoms among the study sample shown by our results exceed those of the general population, which are 0.9% for AN, 1.5% for BN, and 3.5% for binge eating disorder (BED) [40]. The higher incidence of ED symptoms observed in students in comparison with the general population can be explained by referring to the specifics of this age group. Some researchers suggest that starting education, increasing autonomy, stress, social pressure, and low self-esteem may lead to a change in eating behaviors, especially to avoid weight gain, in students [41].

In addition to the student status, gender can be a factor influencing the occurrence of ED [42]. Research findings indicate that AN, BN, and BED are more commonly found among women than men [43,44,45,46]. Similarly, in our study, more women showed the symptoms of ED. Some studies also indicate a higher incidence of ON tendency in women [15,47,48], but this was not confirmed by our results. Women show a specific attitude toward food and body weight due to their desire to achieve or maintain a slim figure [49]. Thus, women give more importance to diet [50]. Nevertheless, in our sample, we observed a higher percentage of women eating meals at the recommended intervals (i.e., every 3–4 h) [51], which confirms their higher concern for diet [52]. In the case of other eating habits, no differences were found between men and women. This can be explained by the greater influence of age (student status) [41] than gender on the eating behaviors of the study sample.

Students who displayed only ED symptoms (“ED and without ON”) were characterized by unhealthy eating behaviors. They consumed less than the recommended number of meals, ate irregularly and with a break of more than 4 h between meals, and skipped dinner and supper. Similar eating habits, including having long breaks between meals and skipping meals [53], have been reported in a study on AN patients with binge eating or purging behavior. In the present study, the relationship between ED symptoms (“ED and without ON”) and the identified dietary patterns was not identified. Therefore, it can be assumed that ED is not associated with the frequency of eating food products but with specific eating habits [54], including following a special diet, as the participants with such eating habits declared the use of special diets both currently and in the past.

Our results indicated that people with ON tendency (“ON and without ED”) showed more positive eating behaviors compared to the other people. A higher percentage of these people were found to eat four or more meals a day, eat meals regularly, have a 3–4 h break between meals, and to not skip dinner and supper. In addition, these people less frequently consumed sugar-rich and refined products than others, and so their diet contained less sugar and salt. According to Barrada and Roncero [38], orthorexia contains two dimensions, i.e., pathological and non-pathological interest in healthy eating. While the pathological dimension of orthorexia is associated to negative affect and the fear of unhealthy eating, non-pathological interest in healthy eating is associated with the choice of healthy food and may serve as a protective factor against emotional distress [55]. Furthermore, orthorexia shares a primary component with restrained eating, i.e., self-imposed restriction of permitted food [56]. Nevertheless, orthorexia is focused on food quality and not quantity [1]. Therefore, the question then arises whether orthorexia should be considered as analogous to restricted nutrition, or as a new style of food. Some researchers suggest that orthorexic eating behavior is a new variant of eating behavior [55]. However, these more adequate eating behaviors can also cause health risks if they get worse and became obsessive. Thus, ON tendency can be seen as desirable (healthy eating) and having high social acceptance [57], but it may also increase the risk of developing ON.

By contrast, people who showed both ON tendency and ED symptoms (“ON and ED”) did not stand out in terms of eating behaviors, except for following a special diet. Similar to people showing only ED symptoms, people who showed both “ED and ON” declared the use of a special diet both currently and in the past. It can be assumed that due to the contrasting eating habits of people showing only ON tendency (“ON and without ED”) and only ED symptoms (“ED and without ON”), no differences were found in the eating habits of people within the group “ON and ED.” In addition, these people were characterized by less frequent consumption of sugar-rich and refined products, which was confirmed by the results of the previous studies on ON [10,26,27] and both AN subtypes—restrictive and binge eating or purging [58,59]. At the same time, people who showed both ON tendency and ED symptoms (“ON and AN”) often consumed meat and meat products (upper tertile of DP). This may be due to the fact that meat products are rich in animal protein that increases only lean body mass and not fat mass [58]. Moreover, the change in eating behaviors seen in people with ED is usually associated with the perceived “fattening” effect [60], which could have contributed to the more frequent consumption of meat and meat products and reduced consumption of sugar-rich and refined products. It can also be assumed that, in people with both ON tendency and ED symptoms, the occurrence of the former was not associated with the use of a vegetarian or vegan diet [56,61].

Among the students who declared that they were currently following a special diet, ON tendency and ED symptoms were found both separately and in combination. Among those who reported the use of a special diet in the past, only the ON tendency (“ON and without ED”) was observed. By contrast, people who did not display ON tendency or ED symptoms (“neither ON nor ED”) were more than 2.5 times more likely to have not followed any diet currently or in the past. Thus, the use of a special diet should be considered as the most important predictor of various types of eating disorders. The use of a special diet both in the past and currently increased the risk of the simultaneous occurrence of ON tendency and ED symptoms to the greatest extent. In addition, this can be associated with concern for the quality of food consumed and the elimination of certain groups of food products [62]. A previous study found that more than half of the ED patients had followed a vegetarian diet in the past, while almost 24% of them are currently vegetarians [63]. In addition, a relationship has been observed between ON tendency and the use of vegetarian and vegan diets [56,61]. Some researchers suggest that vegetarianism and veganism can be a socially acceptable way to avoid certain foods or certain eating situations and used as a mask to hide ED [24,62,63]. However, our results that indicated the frequent consumption of meat and meat products by people with both the ON tendency and ED symptoms (“ON and ED”) suggest that they did not follow a vegetarian diet. Thus, it can be assumed that these people followed other special diets rather than vegetarian or vegan ones.

Dietary patterns that were based on the frequency of food consumption did not show large differences between the groups identified with ON tendency and ED symptoms, except for “high-sugar products and refined products” and “meat and meat products.” However, the usefulness of DPs has been confirmed in previous studies [64,65]. Therefore, the frequency of food consumption may not serve as a good indicator for assessing the nutritional specificity of both disorders. In turn, it is suggested to consider the amount of food consumed to identify the dietary patterns of people displaying disordered eating behaviors in further research. In our study, we did not find any relationship between the occurrence of ED symptoms (“ED and without ON”) and DPs, except for “high-sugar products and refined products.” Our results indicated a higher risk of ON tendency, both with and without ED symptoms, in the group of people rarely consuming these products who represented the bottom tertile of this DP. However, frequent consumption of these products (the upper tertile of this DP) decreased the chances of displaying ON tendency and ED symptoms. Products included in this category have a high content of sugar and salt, which is the reason for their elimination from the diet by people with ON tendency and ED symptoms [9,41].

### Strengths and Limitations

Our study has some limitations that should be acknowledged. ORTO-15 is used by most researchers to identify ON because this tool has been considered as the gold standard for a long time [14]. Nevertheless, it has a few drawbacks with respect to identifying the ON tendency [13]. These include psychometric aspects, such as instability of the internal structure, low internal consistency, uncertainties associated with the scoring system and score interpretation, and limitations of construct validity, as well as the high prevalence of ON tendency [14,18]. According to Dunn and Bratman [2], ORTO-15 does not allow distinguishing between healthy eating and pathological healthy eating. Therefore, some researchers suggest using other tools to measure the ON tendency, such as the Teruel Orthorexia Scale (TOS) [38], the Düsseldorfer Orthorexia Scale (DOS) [66], and the Eating Habits Questionnaire (EHQ) [67]. The psychometric properties of these tools have been tested, showing good internal consistency, good test-retest reliability, and good construct validity [38,66,67]. Therefore, given the validity and reliability of data from these tools, they are good alternative to measuring of ON tendency for ORTO-15 questionnaire [61,68,69]. In spite of its limitations, ORTO-15 is frequently used as it allows comparing the results obtained. For this reason, we used this questionnaire, and it was also used earlier in a study of the Polish population [16].

Because our study sample included students, the results cannot be generalized to the Polish population, or even to people under the age of 35. We focused on students because this group is often shown to experience ON [35,70]. Self-reported data may have also contributed to potential bias when BMI was calculated. Lastly, this was a cross-sectional study and did not allow us to assess the causality of relationships between the variables.

The strength of our study is that it included a large number of students from several universities located in various regions of the country. Moreover, to the best of our knowledge, this study is the first to identify ON tendency and ED symptoms in one study sample and treat participants with individual symptoms and both symptoms separately. In addition, dietary restrictions and the frequency of selected eating habits as well as dietary patterns were used to assess eating behaviors.

## 5. Conclusions

In the present study, students showing ON tendency (without ED symptoms) were found to exhibit more adequate eating behaviors. By contrast, students showing only ED symptoms were characterized by eating behaviors that were more adverse to health. The differences observed in eating behaviors between the two groups confirmed the differences between ON and ED, thus pointing to the need to distinguish them and learn more about their specificity. However, the use of a special diet in the past or currently was associated with both ON tendency and ED symptoms. In addition, both separate and combined symptoms of ON and ED were found to be related to dieting, indicating that following a special diet may increase the risk of eating disorders. The occurrence of ON and the occurrence of ED were associated with specific eating habits. People with ON tendency were more likely to display healthy eating habits, including eating four or more meals a day, regular eating, and no skipping of meals, while the reverse was found in people with ED symptoms. The lack of a relationship between the ON tendency and dietary patterns, with the exception of “high-sugar products and refined products,” and at the same time, more healthy eating habits indicated that the occurrence of ON tendency in the study sample was more closely associated with healthy eating than with the obsession with healthy eating. Moreover, the combined ON tendency and ED symptoms showed no relationship with the eating habits, which is an effect of overlapping of the specifics of both types of eating disorders. This can be a barrier to detecting impaired nutrition because the number of meals consumed, the regularity of meals, and skipping of meals are easy-to-observe indicators of healthy eating. Our findings, especially the coexistence of ON tendency and ED symptoms, provide new information and indicate the direction of future research, but in order to confirm them, this research should be repeated and expanded with more sensitive and validated tools that can better identify ON tendency and ED symptoms. Thus, there is still a need for further research involving the use of more sensitive tools that can better identify ON tendency and ED symptoms, as well as dietary patterns.

## Figures and Tables

**Table 1:** Factor-loading matrix for the dietary patterns (DPs) identified by factor analysis.

Variables	High-Sugar and Refined Products	Meat and Meat Products	Alcohol	High-Fiber Products and Nuts	Dairy Products and Whole-Meal Bread
Sugar	0.555 *	0.117	0.016	−0.120	−0.026
Chocolate, chocolate candies and chocolate bars	**0.619**	−0.020	−0.034	−0.022	0.001
Non chocolate candies	**0.610**	0.082	0.059	0.083	−0.099
Biscuits and cookies	**0.642**	0.006	−0.027	−0.013	0.114
Salty snacks	**0.567**	0.069	0.256	−0.032	−0.121
Sweetened milk drinks	**0.628**	0.044	0.061	−0.012	0.258
Refined bread	**0.600**	0.129	−0.077	−0.244	0.017
Dried fruits	−0.088	−0.050	−0.045	**0.704**	0.136
Dried vegetables	0.066	0.121	0.133	**0.624**	−0.065
Legumes	0.008	−0.052	0.023	**0.643**	0.035
Nuts	−0.190	0.066	−0.025	**0.593**	0.117
Seeds (e.g., pumpkin, sesame, sunflower, wheat germ)	−0.155	−0.043	0.032	**0.638**	0.118
Beer	0.160	0.080	**0.731**	−0.077	−0.056
Red wine	−0.010	−0.022	**0.702**	0.199	0.173
White and rose wine	−0.012	−0.072	**0.715**	0.116	0.187
Vodka and strong liquor	0.108	0.137	**0.721**	−0.041	−0.043
Other animal fats	0.220	**0.574**	0.102	0.205	−0.098
Sausages various types (e.g., luncheon meat, frankfurters, bacon)	0.409	**0.596**	0.057	−0.165	−0.023
High-quality cold cuts (e.g., ham, chicken sirloin, pork loin)	0.300	**0.579**	−0.006	−0.212	0.146
Meat products and offal (e.g., liver, black pudding, headcheese)	0.285	**0.591**	0.094	0.058	−0.096
Red meat (e.g., pork, beef, veal)	0.108	**0.743**	−0.002	−0.001	0.003
Poultry meat and rabbit meat	0.002	**0.586**	0.002	−0.087	0.157
Venison	0.101	**0.511**	0.192	0.246	−0.176
Milk and natural milk drinks	0.212	−0.025	0.048	−0.021	**0.693**
Natural cottage cheese	0.145	0.059	0.051	0.203	**0.610**
Whole-meal bread	−0.086	−0.096	0.005	0.148	**0.528**
Variance Explained (%)	13.6	11.5	11.4	9.4	8.9
Total Variance Explained (%)	54.8				
Kaiser’s Measure of Sampling Adequacy:	0.803				

* factor loadings ≥ 0.5 were bolded for each dietary patterns (factors).

**Table 2:** Sociodemographic characteristics of participants.

Variables		*N* = 1120	%
Gender	Male	331	70.4
	Female	789	29.6
Age	≤19	404	36.1
	20–22	388	34.6
	23–25	253	22.6
	≥26	75	6.7
Place of residence	Village	318	28.4
	Town (≤100.000 inhabitants)	319	28.5
	City (>100.000 inhabitants)	483	43.1
Level of education	Bachelor and engineering degree programs	994	88.7
	Master degree programs	126	11.3
College major	Health related	547	48.8
	Nonhealth related	573	51.2
BMI categories (kg/m^2^)	Underweight (BMI < 18.5)	123	11.0
	Normal weight (18.5 ≥ BMI ≤ 24.9)	817	72.9
	Overweight (25.0 ≥ BMI ≤ 29.9)	154	13.8
	Obesity (BMI ≥ 30.0)	26	2.3

*N*—Number of participants; M—Mean; SD—Standard deviation; BMI—Body mass index.

**Table 3:** Orthorexia nervosa tendency, eating disorders symptoms, and eating habits in the total sample and gender groups.

Variables	Total Sample	Female	Male	Chi-Squared Test	*p*-Value
	*N* = 1120	*N* = 789	*N* = 331		
ON and without ED *n* (%)	171 (15.3)	111 (14.1)	60 (18.1)	2.97	0.085
ED and without ON *n* (%)	422 (37.7)	332 (40.1)	90 (27.2)	22.01	<0.0001
ON and ED *n* (%)	146 (13.0)	108 (13.7)	38 (11.5)	1.01	0.316
Neither ON nor ED *n* (%)	381 (34.0)	238 (30.2)	143 (43.2)	17.66	<0.0001
Number of meals a day *n* (%)					
One to two meals	114 (10.2)	76 (9.6)	38 (11.5)	4.88	0.087
Three meals	321 (28.7)	214 (27.1)	107 (32.3)		
Four or more meals	685 (61.1)	499 (63.3)	186 (56.2)		
Regularity of eating meals *n* (%)					
No	657 (58.7)	462 (58.6)	195 (58.9)	0.01	0.912
Yes	463 (41.3)	327 (41.4)	136 (41.1)		
Breaks between meals *n* (%)					
Less than 3 h	70 (6.3)	51 (6.5)	19 (5.7)	12.72	0.001
3–4 h	771 (68.8)	565 (71.6)	206 (62.3)		
More than 4 h	279 (24.9)	173 (21.9)	106 (32.0)		
Skipping breakfast *n* (%)					
No	959 (85.6)	685 (86.8)	274 (82.8)	3.09	0.079
Yes	161 (14.4)	104 (13.2)	57 (17.2)		
Skipping dinner *n* (%)					
No	1034 (92.3)	726 (92.0)	308 (93.1)	0.35	0.552
Yes	86 (7.67)	63 (8.0)	23 (6.9)		
Skipping supper *n* (%)					
No	984 (87.9)	686 (86.9)	298 (90.0)	2.08	0.150
Yes	136 (12.1)	103 (13.1)	33 (10.0)		
Following a special diet in the past *n* (%)					
No	651 (58.1)	448 (56.8)	203 (61.3)	1.98	0.159
Yes	469 (41.9)	341 (43.2)	128 (38.7)		
Currently following a special diet *n* (%)					
No	979 (87.4)	691 (87.6)	288 (87.0)	0.07	0.792
Yes	141 (12.6)	98 (12.4)	43 (13.0)		

ON–orthorexia nervosa; ED–eating disorders; *N*—number of participants.

**Table 4:** Associations between orthorexia nervosa tendency and eating disorders symptoms by eating habits and dietary patterns (odds ratios with 95% confidence intervals).

Eating Habits/Dietary Patterns	ON and without ED			ED and without ON			ON and ED			Neither ON Nor ED		
	*N* = 171			*N* = 422			*N* = 146			*N* = 381		
	OR	95% CI	*p*-Value	OR	95% CI	*p*-Value	OR	95% CI	*p*-Value	OR	95% CI	*p*-Value
Number of meals a day												
One to two meals	0.24	0.09; 0.61	0.003	2.24	1.47; 3.39	<0.001	0.51	0.22; 1.22	0.131	0.89	0.58; 1.38	0.606
Three meals	0.48	0.30; 0.75	0.001	1.40	1.05; 1.87	0.021	1.21	0.78; 1.87	0.406	0.92	0.69; 1.24	0.924
Four or more meals (ref.)	1	-	-	1	-	-	1	-	-	1	-	-
Regularity of eating meals												
No	0.30	0.20; 0,44	<0.0001	2.35	1.78; 3.09	<0.0001	0.79	0.52; 1.19	0.266	0.86	0.66; 1.12	0.271
Yes (ref.)	1	-	-	1	-	-	1	-	-	1	-	-
Breaks between meals												
Less than three hours	0.83	0.38; 1.79	0.825	0.57	0.23; 1.50	0.267	1.90	0.95; 3.83	0.071	1.23	0.72; 2.10	0.443
More than four hours	0.42	0.25; 0.71	0.001	1.49	1.12; 2.01	0.007	0.83	0.50; 1.37	0.461	1.01	0.74; 1.36	0.983
Three to four hours (ref.)	1	-	-	1	-	-	1	-	-	1	-	-
Skipping breakfast												
No	1.52	0.85; 2.74	0.158	0.76	0.54; 1.09	0.137	1.24	0.67; 2.27	0.498	1.02	0.71; 1.46	0.933
Yes (ref.)	1	-	-	1	-	-	1	-	-	1	-	-
Skipping dinner												
No	6.33	1.54; 26.07	0.011	0.56	0.35; 0.89	0.014	1.79	0.71; 4.53	0.221	0.93	0.58; 1.49	0.756
Yes (ref.)	1	-	-	1	-	-	1	-	-	1	-	-
Skipping supper												
No	2.70	1.23; 5.93	0.013	0.59	0.41; 0.87	0.008	1.51	0.74; 3.08	0.254	1.04	0.70; 1.55	0.840
Yes (ref.)	1	-	-	1	-	-	1	-	-	1	-	-
Following a special diet in the past												
No	0.81	0.56; 1.92	0.290	0.74	0.57; 0.96	0.026	0.34	0.23; 0.52	<0.0001	2.69	1.69; 4.28	<0.0001
Yes (ref.)	1	-	-	1	-	-	1	-	-	1	-	-
Currently following a special diet												
No	0.36	0.23; 0.54	<0.0001	0.40	0.26; 0.62	<0.0001	0.26	0.17; 0.38	<0.0001	2.65	1.69; 4.17	<0.0001
Yes (ref.)	1	-	-	1	-	-	1	-	-	1	-	-
“High-sugar products and refined products” DP												
Bottom tertile	1.66	1.05; 2.64	0.032	0.98	0.72; 1.35	0.931	1.67	0.98; 2.82	0.050	0.66	0.48; 0.90	0.009
Middle tertile	1.27	0.78; 2.04	0.336	1.18	0.86; 1.61	0.291	1.54	0.91; 2.62	0.109	0.65	0.47; 0.89	0.007
Upper tertile (ref.)	1	-	-	1	-	-	1	-	-	1	-	-
“Meat and meat products” DP												
Bottom tertile	1.12	0.72; 1.75	0.616	0.86	0.63; 1.18	0.355	0.58	0.34; 0.98	0.043	1.34	0.98; 1.84	0.071
Middle tertile	0.88	0.55; 1.39	0.583	1.14	0.83; 1.56	0.414	1.01	0.63; 1.62	0.965	0.92	0.67; 1.28	0.619
Upper tertile (ref.)	1	-	-	1	-	-	1	-	-	1	-	-
“Alcohol” DP												
Bottom tertile	1.26	0.79; 1.99	0.909	1.02	0.74; 1.40	0.896	0.72	0.43; 1.20	0.209	0.99	0.72; 1.36	0.973
Middle tertile	1.14	0.72; 1.81	0.567	1.23	0.90; 1.68	0.189	0.88	0.54; 1.43	0.612	0.79	0.58; 1.09	0.159
Upper tertile (ref.)	1	-	-	1	-	-	1	-	-	1	-	-
“High-fiber products and nuts” DP												
Bottom tertile	0.87	0.55; 1.36	0.529	1.14	0.83; 1.56	0.428	1.16	0.71; 1.87	0.558	0.89	0.65; 1.22	0.461
Middle tertile	0.84	0.54; 1.32	0.456	1.34	0.98; 1.83	0.070	0.78	0.46; 1.32	0.347	0.88	0.64; 1.22	0.460
Upper tertile (ref.)	1	-	-	1	-	-	1	-	-	1	-	-
“Dairy products and whole-meal bread” DP												
Bottom tertile	0.91	0.57; 1.45	0.688	0.99	0.73; 1.35	0.959	0.79	0.47; 1.33	0.377	1.15	0.84; 1.59	0.382
Middle tertile	1.15	0.74; 1.75	0.531	0.87	0.63; 1.18	0.364	1.08	0.66; 1.75	0.763	1.05	0.76; 1.44	0.781
Upper tertile (ref.)	1	-	-	1	-	-	1	-	-	1	-	-

Ref., reference group; OR, point estimate (e^β^); (95% CI), 95% Wald Confidence; ON–orthorexia nervosa; ED–eating disorders; DP–dietary patterns.
